# Developmental neurotoxicity of atrazine: decreased synaptic spine density and miRNA-driven GluN2A downregulation in primary hippocampal neurons

**DOI:** 10.1007/s00204-026-04490-0

**Published:** 2026-07-14

**Authors:** Melania Maria Serafini, Agnese Graziosi, Miriam Midali, Luca Ghelli, Giacomo Grumelli, Giulia Sita, Emanuela Corsini, Marina Marinovich, Fabiana Morroni, Barbara Viviani

**Affiliations:** 1 Department of Pharmacological and Biomolecular Sciences “Rodolfo Paoletti”, Università degli Studi di Milano, Milan, Italy; 2 Department of Pharmacy and Biotechnology, Alma Mater Studiorum Università di Bologna, Bologna, Italy; 3 Department of Oncology and Hematology-Oncology, Università degli Studi di Milano, Milan, Italy; 4 Center of Research on New Approach Methodologies (NAMs) in chemical risk assessment (SAFE-MI), Università degli Studi di Milano, Milan, Italy

**Keywords:** Atrazine, Developmental neurotoxicity, Synaptic spines, Glutamatergic system, MicroRNAs

## Abstract

Atrazine (ATZ) is a triazine herbicide and a persistent environmental contaminant. Its use is associated with effects on the nervous system in experimental models. Behavioral and molecular alterations have been reported after exposure, but the cellular mechanisms underlying atrazine-induced developmental neurotoxicity remain unclear, particularly at the synaptic level. In this study, we investigated the effects of ATZ exposure on glutamatergic synaptic maturation in rat primary hippocampal neurons. Neuronal cultures were exposed to ATZ throughout the in vitro developmental period, and structural and molecular endpoints were assessed. Dendritic spines were analyzed by confocal microscopy, and the expression of glutamatergic receptor subunits was evaluated by Western blot and RT-qPCR. In parallel, microRNA expression profiles targeting NMDA receptor subunits were examined to explore post-transcriptional regulatory mechanisms. ATZ exposure induced a selective reduction in dendritic spine density, without detectable changes in spine morphology. This structural effect was accompanied by a selective decrease in the NMDA receptor subunit GluN2A at both protein and mRNA levels, while GluN1, GluN2B, and AMPA receptor subunits were unaffected. Notably, GluN2A downregulation was preceded by the upregulation of microRNAs predicted to target the GRIN2A transcript, indicating a microRNA-mediated regulatory mechanism. Overall, these results demonstrate that ATZ can interfere with hippocampal synaptic maturation by selectively modulating NMDA receptor subunit composition through epigenetic mechanisms. This study identifies dendritic spine and GluN2A regulation as sensitive endpoints of ATZ-induced developmental neurotoxicity and provides mechanistic insight into how early synaptic processes may be affected by this herbicide.

**Supplementary Information:**

The online version contains supplementary material available at https://doi.org/10.1007/s00204-026-04490-0.

## Introduction

Atrazine, ATZ (2-chloro-4-ethylamino-6-isopropylamino-1,3,5-triazine), is a triazine herbicide widely used for the control of broadleaf and grassy weeds. Its use has been banned in Europe since 2004, although it continues to be extensively applied in many other countries, such as the United States, ranking as the second most used herbicide globally (Atwood and Paisley-Jones 2017). As such, human exposure remains a concern due to persistent environmental contamination (Sass and Colangelo 2006). This persistence is mainly related to low biodegradability: its degradation products can stay in the environment for long periods (half-life in water > 200 days; half-life in soil ranging from > 14 to < 109 days) (ATSDR 2024). The most common route for ATZ exposure is oral, through contaminated water and food (EPA 2019). However, occupational exposure may also occur during manufacturing, handling, or application (via inhalation and dermal contact). After absorption, ATZ undergoes metabolism in the liver, which is mediated mainly by cytochrome P450 enzymes; extrahepatic metabolism can also occur in tissues such as the kidney and placenta (Zhao et al. 2021). ATZ metabolites are generally considered to exhibit a toxicological profile comparable to that of their parent compound (WHO, 2011). Physiologically-based kinetic (PBK) modelling studies showed that most ATZ is metabolized and eliminated within 48 h (Joo et al. 2010; Lin et al. 2011). Because it is persistent in the environment, the main concern is continuous exposure rather than bioaccumulation. Rodent studies indicate that ATZ, and its metabolite didealkylatrazine (DACT), crosses the blood–brain and placental barriers, reaching the fetus at maternal plasma concentrations (Ross et al. 2009; Lin et al. 2013). Thus, ATZ exposure can start in utero and persist postnatally, as confirmed by its presence in breast milk (Balduini et al. 2003). This allows ATZ to reach the developing brain during critical windows, potentially interfering with developmental pathways.

In vivo studies have shown that exposure to ATZ during gestation and lactation can alter spatial learning and memory in the offspring (Li et al. 2018; Wang et al. 2019; Honatel et al. 2024), as well as cognitive performance in dams, emotional and motor functions in juvenile offspring, and cognitive ability in adult offspring (Lin et al. 2014), supporting the idea that the developing nervous system is a relevant target of ATZ toxicity. Moreover, structural alterations at the dendritic and synaptic level have been reported, together with deficits in cognition (Yang et al. 2025). The hippocampus is the brain area playing a key role in learning and memory and where glutamatergic N-methyl-D-aspartate (NMDA) receptor- and α-amino-3-hydroxy-5-methyl-4-isoxazolepropionic acid (AMPA) receptor-dependent signaling drives structural and functional refinement of synaptic circuits (Paoletti et al. 2013; Yang et al. 2022). Emerging evidence suggests that ATZ may interfere with these processes. In vivo, chronic ATZ exposure has been associated with changes in NMDA glutamatergic gene expression in the hippocampus (Reyes-Bravo et al. 2022). In vitro, the study by Shan et al. (2021) on human embryonic stem cells at different stages of neural differentiation, indicates altered expression of AMPA glutamatergic markers following ATZ exposure.

Taken together, these data suggest that ATZ may affect hippocampal development through mechanisms that involve, directly or indirectly, the glutamatergic system. However, data from developing hippocampal neurons remain limited, particularly regarding early synaptic processes such as dendritic spine formation and the molecular pathways that regulate their maturation. Our study addresses this gap by examining how ATZ modulates spine development, glutamatergic receptor expression, and microRNA-mediated regulatory pathways in primary hippocampal neurons.

## Materials and Methods

### Primary cultures of rat hippocampal neurons

Primary cultures of rat hippocampal neurons were prepared at embryonic day 18–19 (E18-19). Brains were extracted, the meninges removed, and the cerebral hemispheres separated to isolate hippocampi. Incubation for 5 min at 37 °C in a 2.5% trypsin solution (59427C; Sigma-Aldrich) and DNAsi solution (0.5 mg/ml; DN25; Sigma-Aldrich) allowed for enzymatic digestion. The cell suspension was diluted in Neurobasal medium (21,103–049; Gibco) supplemented with 1% B-27 (17,504–044; Gibco), Penicillin/Streptomycin (ECB3001D; Euroclone), L-glutamine (G7513-100ML; Sigma-Aldrich), and plated onto polyornithine-coated (P3655; Sigma-Aldrich) wells. Cells were seeded at densities and in supports appropriate for each endpoint: 750,000 cells in 60 mm Petri dishes for Western blot analysis; 75,000 cells per coverslip in 12-well plate for transfection and confocal imaging of synaptic spine morphology; 100,000 cells in black 24-well plate for FLUOFORTE® calcium assay. After plating, neuronal cultures were maintained in Neurobasal medium supplemented with 1% B-27 (17,504–044; Gibco), Penicillin/Streptomycin (ECB3001D; Euroclone), and L-glutamine (G7513-100ML; Sigma-Aldrich) under standard conditions (37 °C, 5% CO₂). Cytosine β-D-arabinofuranoside 1 mM (C6645; Sigma-Aldrich) was added to the medium at day in vitro (DIV) 1 to prevent glial cell proliferation. Procedures on rats were approved by the Italian Ministry of Health (authorization n. 5247B.N.XRC, 2020) and were performed according to the European Communities Council Directive 2010/63/EU and the current Italian law on the protection of laboratory animals (D.Lgs. n. 26/2014).

### Atrazine treatments

**Fig. 1:**
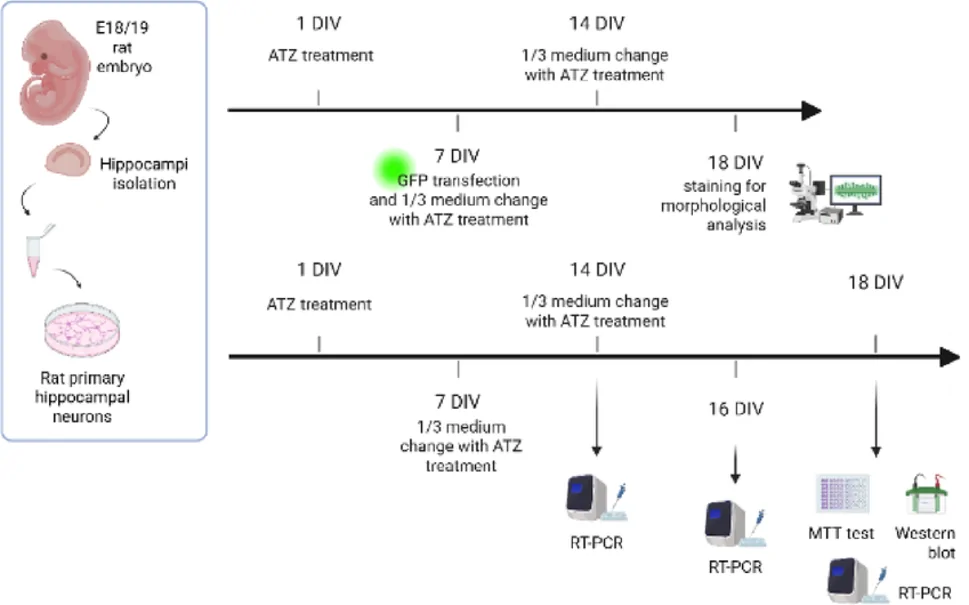
Overview of the in vitro exposure paradigm. Timeline summarizing ATZ treatments and the specific days in vitro at which morphological, molecular, and functional endpoints were collected. *Created in BioRender. Serafini, M. (2026).* https://BioRender.com/wblw5z2

ATZ ≥ 98% purity (45330, Sigma Aldrich, CAS number 1912-24-9) was dissolved in dimethyl sulfoxide (DMSO; A3672; Applichem) and stored at -20 °C in 10 mM stock aliquots. After plating, rat primary hippocampal neurons were treated from DIV 1 to 14, 16, or 18, depending on the analyzed endpoint, to mimic exposure throughout the maturation period. The stock reagent was diluted in culture medium to the working concentration with a final DMSO content of < 0.1% to avoid solvent toxicity. One third of the culture medium was replaced with fresh medium, supplemented with ATZ, once a week (DIV 7 and DIV 14). A schematic overview of the in vitro exposure paradigm is provided in Fig. 1.

### Cell viability

Cell viability was determined using an MTT (M2128, Sigma Aldrich) colorimetric assay, which quantifies mitochondrial dehydrogenase activity by measuring the reduction of 3-(4,5-dimethylthiazol-2-yl)-2,5-diphenyl-tetrazolium bromide to formazan. Cells were seeded in 24-well plates at a density of 50,000 cells per well. After ATZ treatment, 0.75 mg/mL MTT solution was added to each well, and the plate was incubated for 3 h at 37 °C in the dark. A solution of HCl (1 N) and isopropanol (1:24 ratio) was used to dissolve formazan crystals. Absorbance (λ_max_) was measured at 590 nm with a SpectraMax microplate reader (Molecular Devices).

### Transfection, immunofluorescence staining, and confocal imaging for spine morphology analysis

At DIV 7, neurons were transfected with 1.5 µg DNA for green fluorescent protein (GFP) (plasmid kindly provided by Dr. Maria Passafaro) using Lipofectamine 2000 reagent (11668-027; Gibco) following the manufacturer’s instructions. Briefly, the medium was removed from each well and replaced with Neurobasal supplemented with 1% L-glutamine. The same medium was also used to prepare the transfection mix containing DNA and Lipofectamine 2000 reagent. After incubation for 15 min at room temperature (RT), the transfection mix was added dropwise to each well. The plate was then returned to the incubator for 1 h. After the incubation, the transfection medium was removed, and a mixture of conditioned medium (2/3 of the total) and fresh medium supplemented with ATZ (1/3 of the total) was added to each well. At DIV 18, transfected hippocampal neurons were fixed using a solution of 4% paraformaldehyde (1.00496; Sigma-Aldrich) plus 4% sucrose for 10 min at RT. Coverslips were then rinsed with PBS, permeabilized with 0.1% Triton X-100 in PBS for 15 min at RT, and blocked with 5% bovine serum albumin (BSA, A2153, Sigma-Aldrich) in PBS for 1 h at RT. The cells were then incubated with a GFP primary antibody in 5% BSA in PBS for 1 h at RT in a humidified chamber. After four 5-minute PBS washes, the cells were incubated with a fluorophore-conjugated secondary antibody in 5% BSA in PBS for 1 h at RT in a dark, humidified chamber. Following this, the cells were washed with PBS in the dark and mounted onto glass slides. The slides were stored at 4 °C until acquisition. Images were taken with an inverted LSM900 confocal microscope (Zeiss) equipped with a 63× oil objective. Neurons were randomly selected. Images were acquired with the Z-stack mode (Z-step = 0.35 μm; pixel size = 0.09 μm) and then analyzed with ImageJ software. For each neuron (~ 150 μm dendrites), 3 parameters were measured for each spine: length, neck width, and head width. The ratios between the three different measures provide the classification into thin, stubby, or mushroom spines, as described by Bourne and Harris (2008). Morphological analysis of synaptic spine shape was conducted under blinded conditions.

### Western Blotting

Rat primary hippocampal neurons were harvested and homogenized using a glass-glass potter in ice-cold lysis buffer (0.32 M sucrose, 1 mM Hepes, 1 mM MgCl_2_, 1 mM NaHCO_3_, and 0.1 mM PMSF at pH 7.4). The lysate protein content was quantified, then all samples were standardized to a concentration of 1 µg/µL, and 10 µg of protein were loaded into each gel lane. Proteins were separated using denaturing sodium dodecyl-sulphate polyacrylamide gel electrophoresis (SDS-PAGE) and then transferred to nitrocellulose membranes (Bio-Rad). Membranes were blocked for 1 h at RT in I-block solution (I-block T2015, Thermo Fisher Scientific, diluted in Tris-buffered saline plus 1% Tween 20 P1379, Sigma-Aldrich; TBS-T) on a shaker. They were then incubated overnight at 4 °C with primary antibodies in I-block. The next day, after 3 washes with TBS-T, the membranes were incubated with the corresponding horseradish peroxidase (HRP)-conjugated secondary antibodies in I-block for 1 h at RT. After 3 further washes with TBS-T, the membranes were exposed to enhanced chemiluminescence (ECL) reagents (Bio-Rad). To quantify protein expression, the membranes were scanned using a Chemidoc, and the analysis was performed using computer-assisted imaging (Image Lab Software, Bio-Rad). Antibodies used are listed below.

### Antibodies

Table 1 provides all details on the primary and secondary antibodies used for western blotting and immunofluorescence staining

**Table 1:** Antibodies used for Western blotting and immunofluorescence staining

Target	Brand	Product code	Dilution
monoclonal anti-GluN2B	Neuromab	75–097 clone N59/20	1:1,000
monoclonal anti-GluN2A	Sigma Aldrich	M264-10UG	1:1,000
monoclonal anti-GluN1	Invitrogen	32–0500	1:1,000
polyclonal anti-GluA1	Sigma Aldrich	SAB4501293-100UG	1:1,000
monoclonal anti-GluA2	Neuromab	75 − 002 clone L21/32	1:1,000
monoclonal anti-PSD-95	Cell Signaling Technologies	(7E3) 36,233 S	1:1,000
monoclonal anti-actin	Sigma Aldrich	A5441	1:2,500
monoclonal anti-GFP	Neuromab	75–132 clone N86/38	1:300
HRP-conjugated anti-mouse	Bio-Rad	172–1011	1:10,000
HRP-conjugated anti-rabbit	Bio-Rad	172–1019	1:10,000
488-conjugated anti-mouse	Life Technologies	A24350	1:200

### RNA extraction and RT-qPCR

Neurons were harvested, and total RNA extraction was performed by phase separation. Tri-Reagent (T9424, Sigma-Aldrich) was added to each tube, and each sample was vortexed for 1 min. Chloroform (CAS Number 67-66-3, 102447, Merck Millipore) was then added to each tube. The samples were vortexed again for 1 min and placed on ice for 5 min. The samples were then centrifuged at 12,000xg for 15 min at 4 °C. The upper phase from each sample was transferred to a new sterile tube, and pure isopropanol was added (CAS Number 67-63-0, 190764, Sigma Aldrich). Samples were vortexed for one minute and incubated overnight at − 20 °C. The day after, the samples were thawed, vortexed for 1 min, and centrifuged at 12,000xg for 15 min at 4 °C. The supernatant was discarded, and 75% ethanol (CAS Number 64-17-5, 308653, Carlo Erba Reagents) (in RNAse-free water) was added. After further vortexing for 1 min, the samples were centrifuged at 7,600xg for 5 min at 4 °C. The supernatant was removed, and the RNA was dried for 30 min. Finally, RNAse-free water was added, and the samples were boiled at 70 °C for 10 min. RNA concentration of each sample was quantified using a NanoReady Touch F-3100 (LifeReal) instrument. Total RNA samples were stored at -80 °C until further use. For Reverse Transcription quantitative Polymerase Chain Reaction (RT-qPCR), 2 µg of total RNA were retrotranscribed to cDNA with QuantiTect reverse transcription kit (Qiagen) following the manufacturer’s instructions; cDNA was stored at − 20 °C. The amplification reaction was performed using CFX Connect (Bio-Rad), the QuantiTect SYBR green PCR mix (Qiagen), and the QuantiTect Primers (Qiagen). Thermal cycling conditions: 15 min 95 °C (initial activation step), then 40 cycles with 15 s 94 °C, 30 s 55 °C, and 30 s 72 °C. The analysis of mRNA expression was performed using the 2^−ΔΔCt^ method.

### RT-qPCR for microRNA detection

Initially, 100 ng of RNA were retrotranscribed to cDNA using the Mir-X miRNA First-Strand Synthesis and TB Green RT-qPCR (Takara); cDNA was stored at − 20 °C. The assay was run on a Quant Studio 7 Flex real-time PCR system (Applied Biosystems, Thermo Fisher Scientific). Each sample was analyzed in triplicate. Primer sequences were designed using Ensembl (Cunningham et al. 2022) and Benchling; details are reported in Table 2. The relative expression levels were quantified using the 2^− ΔΔCt^ method with miR-U6 as a reference.

**Table 2:** Primer sequences for microRNA detection by RT-qPCR

miRNA	Primer Sequence (5′-3′)
rno-mir-429	TAATACTGTCTGGTAATGCCGT
rno-miR-19a-3p	TGTGCAAATCTATGCAAAACTGA
rno-miR-345-3p	CCCTGAACTAGGGGTCTGGAGA
rno-miR-214-3	ACAGCAGGCACAGACAGGCAG
rno-miR-144-5p	GGATATCATCATATACTGTAAGT
rno-miR-539-5p	GGAGAAATTATCCTTGGTGTGT
rno-miR-200b-3p	TAATACTGCCTGGTAATGATGAC

### Statistical analysis

Prism version 10.2 (GraphPad Software) was used for statistical analyses. Results are presented as mean ± standard error of the mean (SEM). Group comparisons were performed using the appropriate tests (unpaired two-tailed Student’s t-test, one-way ANOVA followed by Tukey or Dunnett post hoc tests); additional details are provided in the figure legends. A significance level of 95% (p-value < 0.05) was set. Values exceeding ± 2.5 standard deviations (SD) from the mean were excluded from the datasets as considered outliers.

## Results

To identify non-toxic ATZ concentrations for our experiments, we assessed cell viability using the MTT assay. Rat primary hippocampal neurons were exposed to increasing concentrations of ATZ (0.02–200 nM) from DIV 1 to DIV 18. As shown in Supplementary Fig. 1, none of the tested concentrations had a significant effect on cell viability.

**Fig. 2:**
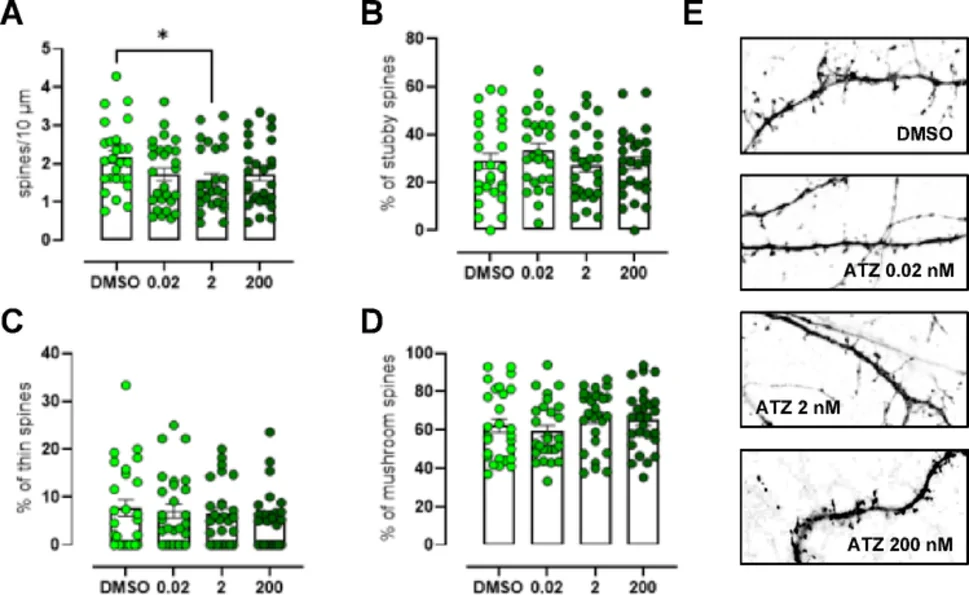
Synaptic spine density and morphology in rat primary hippocampal neurons treated with ATZ. **A** Spine density, defined as the number of spines per 10 µM of analyzed dendrite. **B-D.** Percentage of spines: stubby (**B**), thin (**C**), and mushroom (**D**). **E.** Representative images of DMSO- and ATZ-treated GFP-transfected neurons: dendrites and spines are clearly visible. Dots represent single values, and data are expressed as mean ± SEM (3 neurons randomly chosen from *n* = 9 independent experiments). Measurements have been performed at 18 DIV. One-way ANOVA test followed by Dunnett’s post hoc test. *p < 0.05

**Fig. 3:**
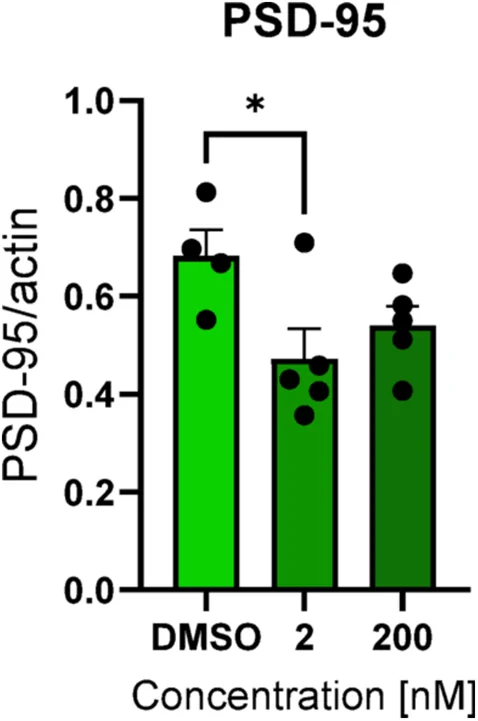
Expression of PSD-95 in rat primary hippocampal neurons exposed to atrazine. Scatter plot representing PSD-95 expression. Data are normalized on the actin levels and expressed as mean ± SEM (*n* = 5). Measurements have been performed at 18 DIV. One-way ANOVA test followed by Dunnett’s post hoc test. *p < 0.05

Based on cell viability data, three concentrations (0.02, 2, and 200 nM) were selected to assess ATZ’s effects on synaptic development during neuronal maturation. Neurons were transfected with GFP to visualize dendritic arborization and quantify synaptic spines. Analysis revealed a significant reduction in spine density at 2 nM compared to control (Fig. 2A), while 0.02 and 200 nM showed no effect. This pattern suggests a non-linear response in this specific endpoint. Spine morphology was unaffected, as the percentages of stubby (Fig. 2B), thin (Fig. 2C), and mushroom (Fig. 2D) spines did not differ from control, as also illustrated by representative images of DMSO- and ATZ-treated GFP-transfected neurons (Fig. 2E). To further confirm the reduction in spine density, we assessed by Western blot the expression of PSD-95, a neuronal protein located exclusively at the postsynaptic site (Vallejo and Inestrosa 2018). As shown in Fig. 3, treatment with 2 nM ATZ resulted in a significant decrease in PSD-95 levels.

**Fig. 4:**
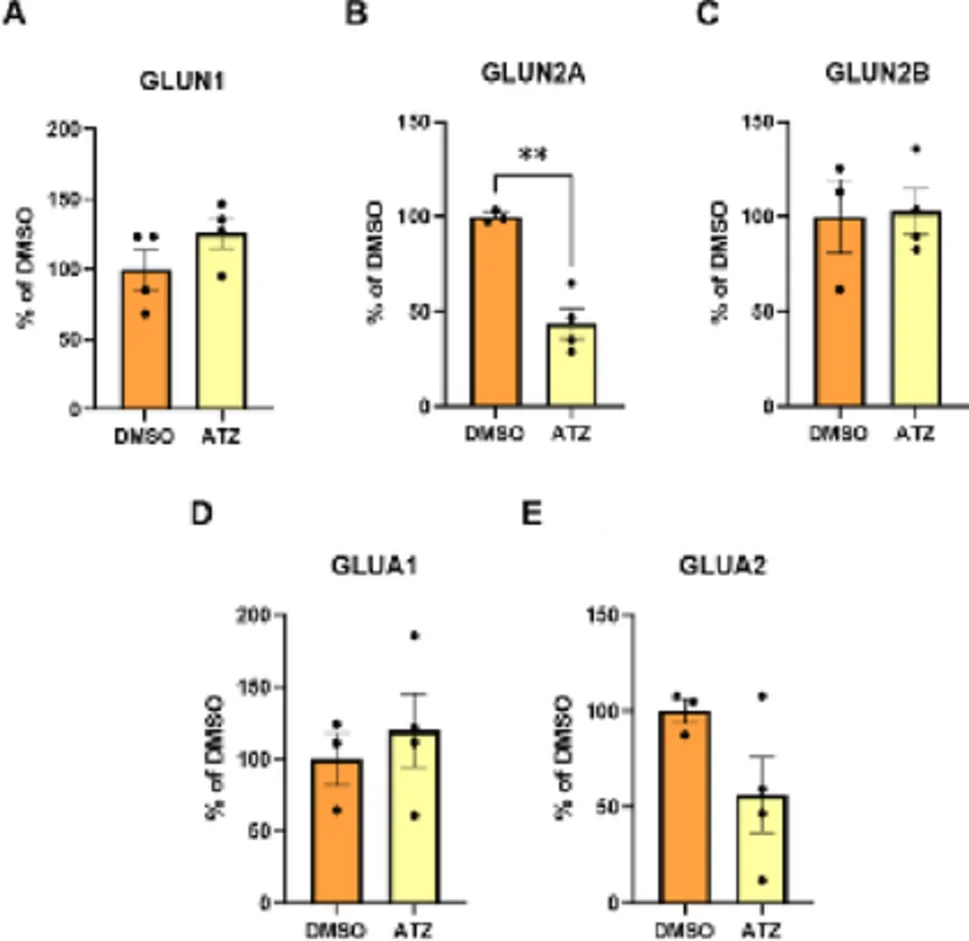
NMDA and AMPA receptor subunit expression in 2 nM atrazine-treated rat primary hippocampal neurons. A-C. Scatter plot representing GluN1 (**A**), GluN2A (**B**), GluN2B (**C**), GluA1 (**D**), and GluA2 (**E**) expression. Data are normalized on the actin levels and expressed as mean ± SEM (*n* = 4). Measurements have been performed at 18 DIV. Unpaired two-tailed Student’s t-test. **p < 0.01

Due to the crucial role of glutamatergic NMDA and AMPA receptors in synaptogenesis, circuit development and plasticity and their relevance in shaping learning and memory (Paoletti et al. 2013), we analyzed their principal subunits by Western blot to determine whether ATZ affects their expression. Based on the previous findings at 2 nM ATZ, we focused on this concentration to proceed with further analysis. Western blot analysis of NMDA receptor subunits revealed a significant reduction in GluN2A expression in neurons treated with 2 nM ATZ compared to controls (Fig. 4B). No significant changes were detected for GluN1 or GluN2B (Fig. 4A and C). Western blot analysis of AMPA receptor subunits showed no significant changes in GluA1 or GluA2 expression following atrazine exposure at 2 nM compared to controls (Fig. 4D and E).

Expression of NMDAR subunits mRNA changes across ontogeny (Law et al. 2003) and some evidence showed that microRNAs are involved in this process (Corbel et al. 2015). Thus, we analyzed the expression of microRNAs predicted to target NMDA receptor subunits (GluN1, GluN2A, and GluN2B). Table 3 reports the microRNAs analyzed in this study along with their predicted NMDA receptor subunit gene targets (GRIN1 for GluN1, GRIN2A for GluN2A, and GRIN2B for GluN2B), identified using the MicroRNA Target Prediction Database (miRDB; https://mirdb.org/), an online resource for miRNA target prediction.

**Table 3:** List of microRNAs analyzed in this study and their predicted NMDA receptor subunit target genes

GRIN1	GRIN2A	GRIN2B
rno-miR-214-3p	rno-miR-19a-3p	rno-miR-345-3p
rno-miR-144-5p	rno-mir-200b-3p	rno-miR-539-5p
	rno-mir-429	

**Fig. 5:**
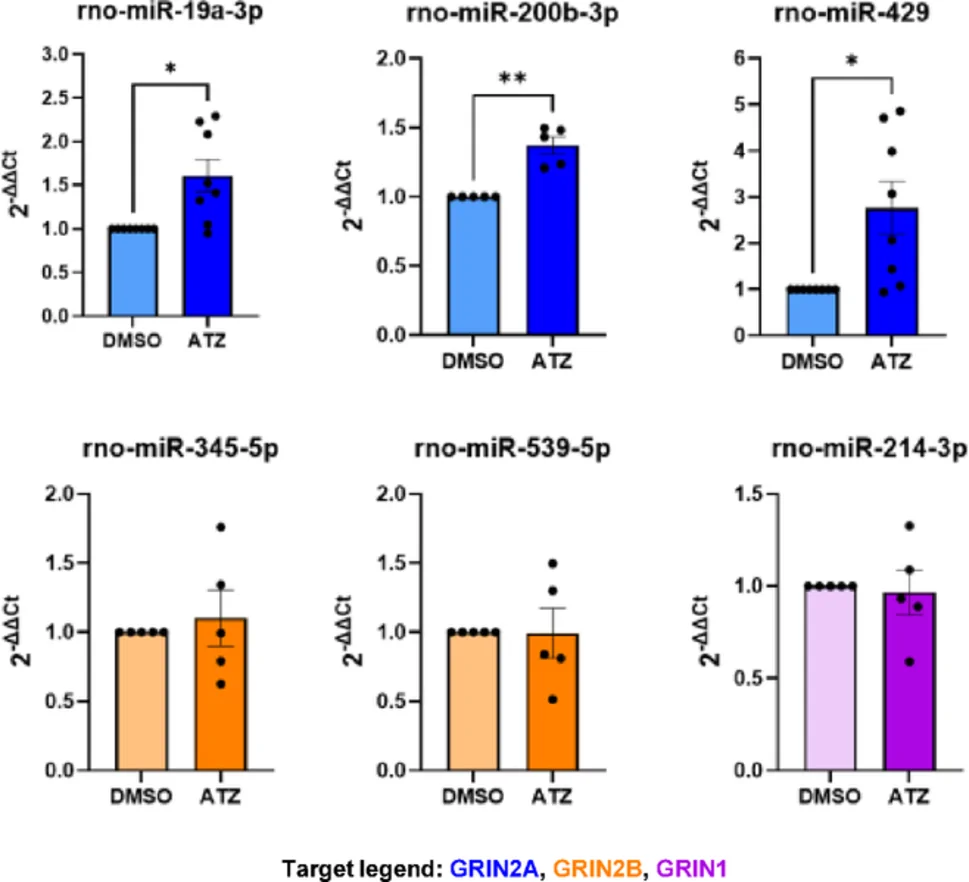
Expression of microRNAs targeting NMDA receptor subunit genes in rat primary hippocampal neurons exposed to atrazine. Data are expressed as fold change relative to control (DMSO) using the 2^−ΔΔCt^ method, normalized to U6. Measurements have been performed at 14 DIV. Data are expressed as mean ± SEM (*n* = 8 independent experiments for rno-miR-19a-3p and rno-miR-429; *n* = 5 for rno-miR-200b-3p, rno-miR-345-3p, rno-miR-539-5p, and rno-miR-214-3p). Statistical analysis: unpaired two-tailed Student’s t-test; *p < 0.05, **p < 0.01. rno-miR-144-5p was not detected in either condition (below detection threshold)

As shown in Fig. 5, ATZ exposure at 2 nM significantly increased the expression of rno-miR-19a-3p (**A**), rno-miR-200b-3p (**B**), and rno-miR-429 (**C**), all targeting GluN2A. In contrast, microRNAs targeting GluN2B (rno-miR-345-3p and rno-miR-539-5p, **D-E**) and GluN1 (rno-miR-214-3p, **F**) showed no significant changes compared to controls. Among all the analyzed microRNAs, rno-miR-144-5p (targeting GluN1) was not detected in either control or ATZ-treated samples, indicating that its expression level was below the detection threshold in our rat primary hippocampal neurons under the aforementioned experimental conditions.

**Fig. 6:**
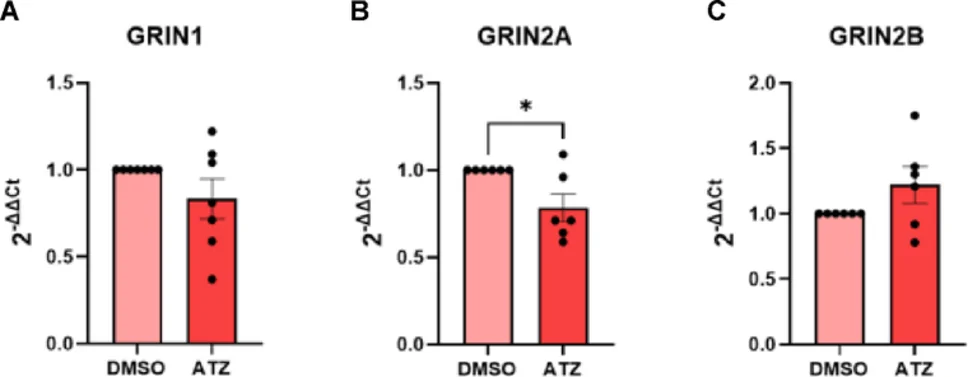
Expression of mRNAs coding for NMDA receptor subunits in rat primary hippocampal neurons exposed to atrazine. Data are expressed as fold change relative to control (DMSO) using the 2^−ΔΔCt^ method, ACTB was the housekeeping gene. Measurements have been performed at 16 DIV. Data are expressed as mean ± SEM (*n* = 7). Statistical analysis: unpaired two-tailed Student’s t-test; *p < 0.05

To validate whether the observed changes in protein expression and microRNA levels were associated with transcriptional modulation, we quantified mRNA expression of GRIN1, GRIN2A, and GRIN2B by RT-qPCR. This longitudinal analysis was performed to verify the biological plausibility of the temporal sequence, determining whether transcriptional changes precede or follow the observed protein and microRNA modulation. As shown in Fig. 6, ATZ exposure at 2 nM significantly reduced GRIN2A mRNA levels at DIV 16, consistent with the decrease observed at the protein level (measured at 18 DIV) and the upregulation of microRNAs targeting GRIN2A (measured at 14 DIV). In contrast, GRIN1 and GRIN2B mRNA expression remained unchanged at that time point. No significant changes were detected for any of the analyzed mRNAs at DIV 14 (Supplementary Fig. 2) and DIV 18 (Supplementary Fig. 3).

## Discussion

Tissue distribution studies indicate that ATZ and its metabolites reach brain concentrations comparable to, or even higher than, blood levels (Brandhonneur et al. 2020; Simpkins et al. 2026). Specifically, in vivo studies in pregnant rats demonstrated that ATZ crosses the placental and blood–brain barriers, with maternal brain concentrations exceeding plasma values (Brandhonneur et al. 2020). Physiologically based pharmacokinetic (PBK) modeling predicts that continuous exposure to 1 mg/kg/day ATZ results in plasma levels of approximately 1 nM (Campbell et al. 2016).

Our findings indicate that ATZ, at concentrations not affecting neuronal viability, can interfere with hippocampal glutamatergic synaptic development. Exposure to 2 nM ATZ significantly reduced spine density without affecting spine morphology, suggesting a selective impact on synaptogenesis rather than on spine maturation. This effect was accompanied by a specific downregulation of the NMDA receptor subunit GluN2A at both protein and mRNA levels, while GluN1, GluN2B, and AMPA receptor subunits remained unchanged. The reduction in GluN2A is preceded by the upregulation of microRNAs predicted to target GRIN2A, supporting a post-transcriptional regulatory mechanism. The pattern of microRNA modulation induced by 2 nM ATZ is consistent with the selective effect on NMDAR subunits, since only microRNAs targeting GRIN2A are upregulated, while those modulating GRIN1 or GRINB remain unaffected. Indeed, epigenetic dysregulation is a documented mode of action for ATZ (Zhao et al. 2024; Arabi et al. 2024). Graziosi et al. (2022) demonstrated that ATZ significantly modulates microRNA expression in differentiated SH-SY5Y human neuronal cells, and recent in vivo findings confirmed that miRNA dysregulation is a central feature of ATZ neurotoxicity in dopaminergic regions (Chen et al. 2025; Li et al. 2025). Our data extend this concept to the hippocampal area. Although transcriptomic alterations have been described in hippocampal progenitor cells exposed to concentrations of ATZ in the micromolar range (Li and Bi 2023), our results show that miRNA modulation also occurs at nanomolar concentrations in rat primary hippocampal neurons.

The selective reduction in total GluN2A provides a plausible molecular basis for the structural synaptic impairments observed in our study. The study from Li et al. (2019) administered ATZ (10 or 100 mg/kg body weight) for 30 days to postnatal day 35 rats demonstrating that ATZ suppressed the MEK/ERK/CREB pathway, leading to a reduced expression of key plasticity markers, such as the Brain Derived Neurotrophic Factor (BDNF). GluN2A-containing NMDAR activation is an upstream trigger for ERK phosphorylation and CREB-mediated gene transcription (Franchini et al. 2020; Ma et al. 2023). The GluN2A downregulation observed may thus be consistent with reduced engagement of ERK/CREB‑dependent pathways regulating BDNF expression, potentially contributing to the synaptic structural alterations observed. Because NMDAR subunit composition influences the efficiency of coupling between calcium entry and downstream signaling (Barria & Malinow 2005), reduced GluN2A availability may compromise GluN2A-dependent pathways required for the stabilization of developing spines, offering a potential explanation for the observed spine loss.

Taken together, these observations highlight the specificity of the effect on GluN2A, a subunit that plays a critical role in hippocampal synaptic development. As far as we are aware, the effects of ATZ on dendritic spine structure in hippocampal neurons have not been explored in vitro. Our findings provide a plausible structural substrate for alterations in spatial learning and memory in the offspring of ATZ-exposed dams (Li et al. 2018; Wang et al. 2019; Honatel et al. 2024) and for the hippocampus-dependent learning and memory impairments recently reported in juvenile mice (Zhu et al. 2023). Our molecular results partially are in line with those of Wang et al. (2019), who also reported reduced PSD-95 levels in the offspring of ATZ-exposed dams at oral maximum tolerated dose (ATZ 100 mg/kg). While Wang et al. (2019) do not evaluate GluN2A, they observed a decrease in GluN2B. Recent data on chronic exposure in adult rats showed the mRNA upregulation of the obligate NMDA subunit GRIN1 in the hippocampus at doses comparable to our model (1 mg/kg day, corresponding to plasma levels of approximately 1 nM) (Reyes-Bravo et al. 2022; Campbell et al. 2016). This pattern is consistent with broader alterations in glutamatergic homeostasis reported in other brain regions and described by Chávez-Pichardo et al. (2020), who showed that chronic exposure to 1 mg/kg ATZ alters glutamate and glutamine levels in regions such as the prefrontal cortex and amygdala. Evidence from human-derived cells further supports the susceptibility of the glutamatergic system: Shan et al. (2021) reported that ATZ exposure during human embryonic stem cell differentiation dysregulated the mRNA expression of the AMPA receptor subunit GRIA1. Despite the high concentrations used (100 µM), their findings still indicate that the glutamatergic system represents a conserved target of ATZ toxicity (Shan et al. 2021). All together these results suggest that ATZ impacts the glutamatergic system differentially, both in vivo and in vitro, depending on the dose/concentration of exposure, the time window (development or adult) and the brain area. Our experimental timeline (DIV 1–18) covered the critical developmental GluN2B to GluN2A switch. The preservation of GluN2B expression, a subunit abundantly expressed during early developmental stages, together with the selective reduction of GluN2A suggests that prolonged ATZ exposure interferes with the normal maturation of NMDA receptor composition, rather than causing a generalized loss of NMDA receptor subunits.

These findings indicate that ATZ can subtly reshape synaptic architecture and NMDA receptor composition during development, with potential consequences for plasticity and circuit refinement.

The observation that ATZ reduced synaptic spine density at 2 nM, but not at lower or higher concentrations, suggests a potentially non-linear response within this experimental system. This pattern is restricted to a single endpoint; therefore, it cannot be generalized and should be interpreted with caution. While similar non-linear responses to ATZ have been reported in vivo, these studies were primarily conducted in non-mammalian models and involved endpoints unrelated to neurotoxicity (Brodeur et al. 2013; Shenoy et al. 2014). Yang et al. (2025) described a non-linear effect of ATZ in the expression of mitochondrial regulatory genes in vitro using the HT22 immortalized mouse hippocampal neuronal cells. However, further studies are required to determine the reproducibility and biological significance of this finding. The biological relevance of 2 nM ATZ is further supported by the consistency of its effect across multiple, mechanistically related endpoints within the same experimental system. Taken together, these findings suggest that ATZ can interfere with hippocampal synaptic maturation through epigenetic modulation of NMDA receptor expression, specifically via miRNA-mediated downregulation of the GluN2A subunit.

## Supplementary Information

Below is the link to the electronic supplementary material.

Supplementary Material 1

## Data Availability

The data are available upon request to the corresponding author.
