# Supplementary material for: Developmental neurotoxicity of atrazine: decreased synaptic spine density and miRNA-driven GluN2A downregulation in primary hippocampal neurons
Source: Arch Toxicol. 2026 Jul 14;100(12):5643–51. doi: 10.1007/s00204-026-04490-0 (PMC13748268; doi:10.1007/s00204-026-04490-0)
Supplement: Supplementary file 1 [file 204_2026_4490_MOESM1_ESM.docx]

**Supplementary figures**

**Supplementary Figure 1. Cell viability of rat primary hippocampal neurons treated with ATZ.** Data are expressed as % of DMSO and shown as mean ± SEM (n=4). One-way ANOVA test followed by Dunnett’s post hoc test.

**Supplementary figure 2. Expression of mRNAs coding for NMDA receptor subunits in rat primary hippocampal neurons exposed to atrazine.** Data are expressed as fold change relative to control (DMSO) using the 2^^−ΔΔCt^ method, ACTB was the housekeeping gene. Measurements have been performed at 14 DIV. Data are expressed as mean ± SEM (n = 7). Statistical analysis: unpaired two-tailed Student’s t-test; **p* < 0.05.

**Supplementary figure 3. Expression of mRNAs coding for NMDA receptor subunits in rat primary hippocampal neurons exposed to atrazine.** Data are expressed as fold change relative to control (DMSO) using the 2^^−ΔΔCt^ method, ACTB was the housekeeping gene. Measurements have been performed at 18 DIV. Data are expressed as mean ± SEM (n = 7). Statistical analysis: unpaired two-tailed Student’s t-test; **p* < 0.05.
